# Cardiac‐specific ablation of glutaredoxin 3 leads to cardiac hypertrophy and heart failure

**DOI:** 10.14814/phy2.14071

**Published:** 2019-04-29

**Authors:** Jimmonique Donelson, Qiongling Wang, Tanner O. Monroe, Xiqian Jiang, Jianjie Zhou, Han Yu, Qianxing Mo, Qin Sun, Juan C. Marini, Xinquan Wang, Paul A. Nakata, Kendal D. Hirschi, Jin Wang, George G. Rodney, Xander H.T. Wehrens, Ninghui Cheng

**Affiliations:** ^1^ USDA/ARS Children Nutrition Research Center Department of Pediatrics Baylor College of Medicine Houston Texas; ^2^ Molecular Physiology & Biophysics Baylor College of Medicine Houston Texas; ^3^ Pharmacology and Chemical Biology Baylor College of Medicine Houston Texas; ^4^ Molecular and Cellular Biology Baylor College of Medicine Houston Texas; ^5^ Ministry of Education Key Laboratory of Protein Science Center for Structural Biology School of Life Sciences Tsinghua University Beijing China; ^6^ Department of Biostatistics & Bioinformatics H. Lee Moffitt Cancer Center & Research Institute Tampa Florida; ^7^ Molecular and Human Genetics Baylor College of Medicine Houston Texas; ^8^ Section of Critical Care Medicine Department of Pediatrics Baylor College of Medicine Houston Texas; ^9^ Center for Drug Discovery Dan L. Duncan Cancer Center Baylor College of Medicine Houston Texas; ^10^ Cardiovascular Research Institute Baylor College of Medicine Houston Texas

**Keywords:** Calcium handling, cardiac hypertrophy, glutaredoxin, heart failure, oxidative stress

## Abstract

Growing evidence suggests that redox‐sensitive proteins including glutaredoxins (Grxs) can protect cardiac muscle cells from oxidative stress‐induced damage. Mammalian Grx3 has been shown to be critical in regulating cellular redox states. However, how Grx3 affects cardiac function by modulating reactive oxygen species (ROS) signaling remains unknown. In this study, we found that the expression of Grx3 in the heart is decreased during aging. To assess the physiological role of Grx3 in the heart, we generated mice in which Grx3 was conditionally deleted in cardiomyocytes (Grx3 conditional knockout (CKO) mice). Grx3 CKO mice were viable and grew indistinguishably from their littermates at young age. No difference in cardiac function was found comparing Grx3 CKO mice and littermate controls at this age. However, by the age of 12 months, Grx3 CKO mice exhibited left ventricular hypertrophy with a significant decrease in ejection fraction and fractional shortening along with a significant increase of ROS production in cardiomyocytes compared to controls. Deletion of Grx3 also impaired Ca^2+^ handling, caused enhanced sarcoplasmic reticulum (SR) calcium (Ca^2+^) leak, and decreased SR Ca^2+^ uptake. Furthermore, enhanced ROS production and alteration of Ca^2+^ handling in cardiomyocytes occurred, prior to cardiac dysfunction in young mice. Therefore, our findings demonstrate that Grx3 is an important factor in regulating cardiac hypertrophy and heart failure by modulating both cellular redox homeostasis and Ca^2+^ handling in the heart.

## Introduction

Cardiac hypertrophy can be either an adaptive or a maladaptive response to physiological or pathological stimuli (Molkentin and Dorn 2001). Pathological cardiac hypertrophy occurs in response to various conditions, such as chronic pressure overload, valvular disease, or mechanical stress (Molkentin and Dorn 2001; Hardt and Sadoshima 2004). Although initially beneficial, reinduction of fetal gene expression and continuous activation of stress signaling pathways in cardiomyocytes eventually contribute to contractile dysfunction (Molkentin and Dorn 2001). Prolonged cardiomyocyte hypertrophy leads to the development of overt heart failure and subsequent death (Hilfiker‐Kleiner et al. 2006). Among many local and systemic factors, aging itself is a major risk factor for cardiac dysfunction, including cardiac hypertrophy and heart failure (Dai et al. 2012).

Reactive oxygen species (ROS) can be formed as by‐products of mitochondrial oxidative phosphorylation in the heart (Droge 2002; Cave et al. 2006). ROS can also be actively generated through various oxidases, such as NADPH oxidase, in cardiovascular tissues (Cave et al. 2006). Low/moderate levels of ROS produced by NADPH oxidases have been proven to be essential for normal cardiac growth and function (Prosser et al. 2011). However, excess ROS or a decrease of antioxidant systems leads to oxidative damage (Tsutsui et al. 2011), causing a wide range of damage to macromolecules, and eventually apoptotic or necrotic cell death, which are often associated with human heart diseases (Sawyer et al. 2002).

Thioredoxin (Trx) and glutaredoxin (Grx) are the two major antioxidant systems that play a vital role in redox homeostasis in the cardiovascular system (Yamawaki et al. 2003; Lillig et al. 2008). Grxs are ubiquitous, small heat‐stable disulfide oxidoreductases, which are conserved in both prokaryotes and eukaryotes (Lillig et al. 2008). Grxs can be categorized into dithiol Grxs, which contain two cysteine residues in their active motifs, and monothiol Grxs, which contain a single cysteine residue in their putative motifs (Lillig et al. 2008). There is a growing body of evidence that monothiol Grxs may have multiple functions in the biogenesis of iron–sulfur clusters, iron trafficking and homeostasis, protection of protein oxidation, cell growth, and proliferation (Herrero and de la Torre‐Ruiz 2007). In mammals, there are two monothiol Grxs, Grx3 and Grx5 (Isakov et al. 2000). Grx5 is localized in the mitochondria, and plays a critical role in iron–sulfur cluster biogenesis and heme synthesis in red blood cells (Camaschella et al. 2007) and is crucial for protecting osteoblasts from oxidative stress‐induced apoptosis (Linares et al. 2009). Grx3, also termed thioredoxin‐like 2 (Txnl2) or PICOT (protein kinase C interacting cousin of thioredoxin), interacts with the protein kinase C theta isoform (Isakov et al. 2000). Previous studies demonstrate that Grx3 is essential for early embryonic growth and development (Cha et al. 2008; Cheng et al. 2011). Recent reports also revealed that forced expression of *Grx3* in transgenic rat heart could enhance cardiomyocyte contractility by modulating calcineurin–NFAT‐mediated signaling and PKC*ζ* activity in the progression of pressure‐overload‐induced heart hypertrophy (Jeong et al. 2006, 2008; Oh et al. 2012). A single *Grx3* allele deletion only caused subtle hypertrophic growth after transverse aortic constriction (TAC), which reversed quickly upon removal of the constriction (Cha et al. 2008). While there is growing evidence of a role for Grx3 in the heart, the precise function of Grx3 in the heart remains to be fully elucidated.

In this study, we investigated the expression of Grx3 in the heart at different ages. We characterized the Grx3 CKO mice and revealed that Grx3‐deficient mice developed late onset cardiac hypertrophy and heart failure. We further documented the molecular mechanism underlying Grx3 function in cardiomyocytes. Taken together, these findings suggest that the presence of Grx3 may be critical for maintaining redox homeostasis and proper Ca^2+^ signaling in the cardiomyocytes.

## Materials and Methods

### Reagents

All chemicals were purchased from Sigma‐Aldrich (St. Louis, MO) unless stated otherwise. Anti‐GAPDH was bought from Chemicon International, Inc. Grx3 monoclonal antibody was made in‐house using full‐length human Grx3 recombinant protein. This antibody was validated and used in our previous studies (Cheng et al. 2011; Qu et al. 2011; Pham et al. 2017).

### Animals

A Grx3 floxed mouse strain was generated as described (Pham et al. 2017). The *αMHC*‐Cre mouse strain was obtained from the Schneider Lab (Agah et al. 1997). Grx3 cardiac‐specific knockout (Grx3 CKO) mice were generated by intercrossing Grx3 floxed mice with *αMHC*‐Cre transgenic ones. All lines were backcrossed for at least six generations. All mouse strains were housed in temperature‐controlled environment and fed a standard chow diet (LabDiet 5V5R chow; LabDiet, St. Louis, MO) ad libitum unless stated otherwise. Male mice were used in this study and Grx3 floxed mouse (Grx3^*flox/flox*^) littermates were used as controls. All studies were performed according to protocols approved by the Institutional Animal Care and Use Committee of Baylor College of Medicine conforming to the Guide for the Care and Use of Laboratory Animals published by the U.S. National Institutes of Health (NIH Publication No. 85–23, revised 1996).

### Echocardiography

Lightly anesthetized mice (1.5–2.0% isoflurane) were imaged in the left lateral decubitus position with a linear 30‐MHz probe (VisualSonics Vevo 2100 Imaging System, Baylor College of Medicine Mouse Phenotyping Core, Houston, TX) (Quick et al. 2017). Digital images were collected at a frame rate of 180 images/s. Two‐dimensional images were recorded in parasternal long‐ and short‐axis projections with guided M‐mode recordings. All measurements were done blinded to genotypes.

### Mouse ventricular myocyte isolation and confocal Ca^2+^ imaging

Mouse ventricular myocyte isolation and confocal Ca^2+^ imaging were performed, as previously described (Wang et al. 2015, 2018). In brief, hearts were excised and collected from anesthetized mice and rinsed in Tyrode solution without Ca^2+^ (137 mmol/L NaCl, 5.4 mmol/L KCl, 1 mmol/L MgCl_2_, 5 mmol/L HEPES, 10 mmol/L glucose, 3 mmol/L NaOH, pH 7.4). Hearts were cannulated through the aorta and perfused using a Langendorff system with the same Tyrode solution for 5 min at 37°C, followed by the Tyrode solution containing 20 *μ*g/mL Liberase (Roche, Indianapolis, IN). After digestion for 10 to 15 min at 37°C, hearts were removed and rinsed with 5‐mL KB solution (90 mmol/L KCl, 30 mmol/L K_2_HPO_4_, 5 mmol/L MgSO_4_, 5 mmol/L pyruvic acid, 5 mmol/L *β*‐hydroxybutyric acid, 5 mmol/L creatine, 20 mmol/L taurine, 10 mmol/L glucose, 0.5 mmol/L EGTA, 5 mmol/L HEPES, pH 7.2), minced in KB solution and agitated, then filtered through a 210‐mm polyethylene mesh. The isolated ventricular myocytes were washed once and stored in KB solution at room temperature before use. For confocal Ca^2+^ imaging, ventricular myocytes were incubated with 2 mmol/L Fluo‐4‐acetoxymethyl ester (Fluo‐4AM, Invitrogen, Carlsbad, CA) in normal Tyrode (NT) solution containing 1.8 mmol/L Ca^2+^ for 1 h at room temperature, followed by 15‐min incubation with dye‐free NT solution for de‐esterification and loaded on a laser scanning confocal microscope (LSM 510, Carl Zeiss, Thornwood, NY). Fluorescence images were recorded in line‐scan mode with 1024 pixels per line at 500 Hz. After being paced at 1 Hz for 2 min, only rod‐shaped myocytes showing clear striation and normal contractility were selected for further experiments. Once steady‐state Ca^2+^ transient was observed, pacing was stopped and Ca^2+^ sparks were analyzed. Caffeine (10 mmol/L) was used to induce caffeine‐induced Ca^2+^ transient to calculate sarcoplasmic reticulum (SR) Ca^2+^ content.

### ROS production assay

Dihydroergotamine (DHE) staining to measure ROS production in cardiomyocytes was followed as per published procedures with modification (Pham et al. 2017). In brief, paraffin‐embedded heart sections were immersed in xylene twice for 10 min, in 100% ethanol twice for 10 min, in 95% ethanol once for 5 min, in 75% ethanol once for 5 min, in 50% ethanol once for 5 min, and finally in distilled water once for 10 min. Slides were washed twice for 10 min each in 1× phosphate‐buffered saline (PBS) and fixed in ice‐cold 4% paraformaldehyde (PFA) with PBS for 10 min. After being washed with PBS 3 × 10 min, the slides were treated with 5% Triton X‐100 for 5 min and then washed twice with PBS for 10 min each. The slides were stained with 5 *μ*mol/L DHE for 1 h at room temperature (RT) and washed 3× 10 min with PBS. After staining, the sections were mounted with Vectashield mounting media (Vector Laboratories, Burlingame, CA) and allowed to dry overnight at RT in a cabinet away from light. For DHE staining of myocytes, isolated myocytes were fixed with 4% PFA for 30 min at RT and washed 3 × 5 min with PBS, then stained with 5 *μ*m DHE for 30 min at RT. After staining, cells were washed 5 × 5 min with PBS and mounted. The DHE fluorescence signals were detected at 610 nm (excitation at 520 nm) for using an Olympus Fluoview FV1000 confocal laser scanning microscopy system. The fluorescent intensity was quantified using Image J.

### Quantitative RT‐PCR

Total RNA was extracted from either heart (ventricle) tissues or cardiomyocytes from Grx3 CKO mice and wild‐type (WT) controls using Trizol reagent (Thermo Fisher Scientific, Waltham, MA). Purified RNA samples underwent reverse transcription to yield cDNA. Quantitative polymerase chain reaction (qPCR) was performed using the SYBR Green‐based assay on Bio‐Rad CFX96™ Real‐Time PCR Detection System. Differences in gene expression of control and knockout (KO) mice were quantified by the comparative CT method. Gapdh was selected to serve as the internal control because its expression level was consistent between the WT and KO and relatively stable across different stages.

### Western blot analysis

Heart (ventricle) lysates were prepared from flash‐frozen mouse hearts as described (Wang et al. 2015) and run on sodium dodecyl sulfate‐polyacrylamide gel electrophoresis (SDS‐PAGE) gels. Western blot analysis was conducted following an established procedure (Cheng et al. 2011). Antibodies against GRX3 and GAPDH were used at 1:1000 and 1:2000 dilution, respectively.

#### Statistical analysis

All results were shown as means ± SEM. A two‐way analysis of variance (ANOVA) was used to analyze data presented in Table 1. Student's *t* test was used to compare the two genotypes. *P <* *0.05, **P <* *0.01, and ***P <* *0.001 were used as indicators of the level of significance.

**Table 1:** Echocardiographic analysis of chow diet fed mice at age of 3 and 5 months

	3 months old		5 months old	
	Grx3^*flox/flox*^	Grx3 CKO	Grx3^*flox/flox*^	Grx3 CKO
	*n* = 8	*n* = 15	*n* = 9	*n* = 7
HR	472.74 ± 41.04	485.66 ± 47.51	495.68 ± 49.6	469.14 ± 52.92
LVEED (mm)	4.19 ± 0.31	4.04 ± 0.27	3.77 ± 0.27	4.03 ± 0.29
LVESD (mm)	2.81 ± 0.44	2.76 ± 0.32	2.43 ± 0.35	2.75 ± 0.31
DP WT (mm)	0.69 ± 0.08	0.69 ± 0.1	0.88 ± 0.09	0.83 ± 0.21
SP WT (mm)	1.11 ± 0.08	1.1 ± 0.09	1.36 ± 0.16	1.24 ± 0.16
EF	60.9 ± 8.3	59.2 ± 8.1	65.87 ± 8.44	57.69 ± 8.11
%FS	32.5 ± 5.5	31.2 ± 5.0	35.98 ± 6.31	30.14 ± 5.58
BW (g)	28.58 ± 2.35	26.99 ± 2.87	33.66 ± 2.86	36.52 ± 4.68
HW (mg)	137.2 ± 10.2	137.68 ± 30.64	190.33 ± 44.93	191.57 ± 41.4
HW/BW (mg/g)	4.82 ± 0.39	5.16 ± 1.12	5.76 ± 1.72	5.33 ± 1.42
HW/TL (mg/mm)	8.29 ± 0.39	8.74 ± 0.49	10.52 ± 2.53	10.49 ± 2.34

## Results

### Age‐dependent reduction of Grx3 expression in the heart

**Figure 1:**
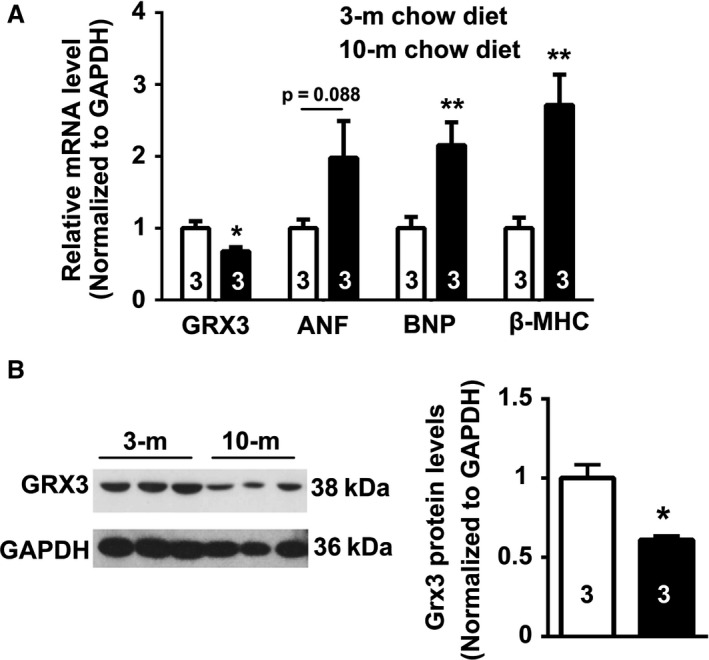
Age‐dependent reduction of Grx3 expression in the heart. (A) Quantitative polymerase chain reaction (q‐PCR) analysis showing reduced *Grx3* mRNA levels in the heart of 10‐month‐old mice compared to 3‐month‐old mice. Student's *t* test, *n* = 3, *P < 0.05, 10‐m versus 3‐m. (B–C) Reduced Grx3 protein levels in older mice (10‐m vs. 3‐m). Student's *t* test, *n* = 3, *P < 0.05, 10‐m versus 3‐m.

To determine whether *Grx3* expression is altered during aging, total RNA samples and tissue homogenates were extracted from hearts of 3‐month‐old and 10‐month‐old mice, respectively. Quantitative RT‐PCR analysis indicated that *Grx3* mRNA levels were significantly downregulated in hearts of 10‐month‐old compared with 3‐month‐old mice (Fig. 1A). In contrast, the expression of cardiac fetal genes was significantly increased in the 10‐month‐old mice (Fig. 1A). Similarly, the levels of Grx3 proteins were reduced in the heart from 10‐month‐old mice compared to the younger ones (Fig. 1B–C).

### Disruption of Grx3 in the mouse heart

**Figure 2:**
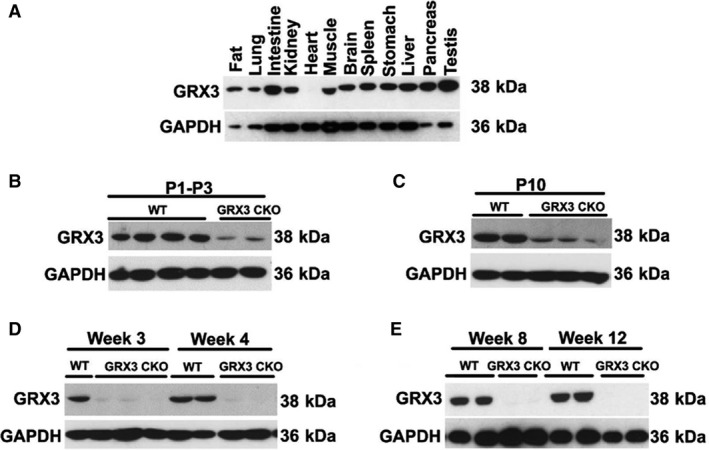
Tissue‐specific deletion of Grx3 in hearts of Grx3 conditional knockout (CKO) mice. (A) Western blot analysis of Grx3 expression (protein level) in different tissues of *α* MHC‐Cre/Grx3^*flox/flox*^ mice. (B–E) Western blot analysis of Grx3 expression in the hearts of day 1 to day 3 postnatal wild‐type (WT) and CKO mice (B), at postnatal day10 (C), at 3 and 4 weeks of age (D), and at 8 and 12 weeks of age (E).

A cardiac‐specific *Grx3*‐deficient mouse line (Grx3 CKO) was created by crossing *Grx3* floxed mice with *α‐Mhc*‐Cre mice. Western blotting revealed that Grx3 expression was selectively disrupted in the heart, while Grx3 levels were unaltered in other tissues (Fig. 2A). Grx3 expression levels were significantly reduced in neonatal CKO hearts compared to littermate controls (Grx3^*flox/flox*^), although residual expression remained (Fig. 2B–C). Starting at the age of weaning, Grx3 protein was completely absent in the hearts of CKO mice (Fig. 2D–E).

### Grx3 deletion mice develop cardiac hypertrophy and heart failure

Deletion of Grx3 in the heart did not affect the growth of CKO mice compared to littermate controls, there was no significant difference in body weight, heart weight, heart‐to‐body weight ratio, and heart weight‐to‐tibial length ratio comparing Grx3 CKO mice and littermate controls at young ages (both 3 and 5 months) (Table 1). Assessment of left ventricular (LV) function by echocardiography revealed no significant differences in cardiac contractility or dimensions, even though there was slight decline in LV ejection fraction and fractional shortening of Grx3 CKO mice compared to littermate controls at 5 months of age (Table 1).

**Figure 3:**
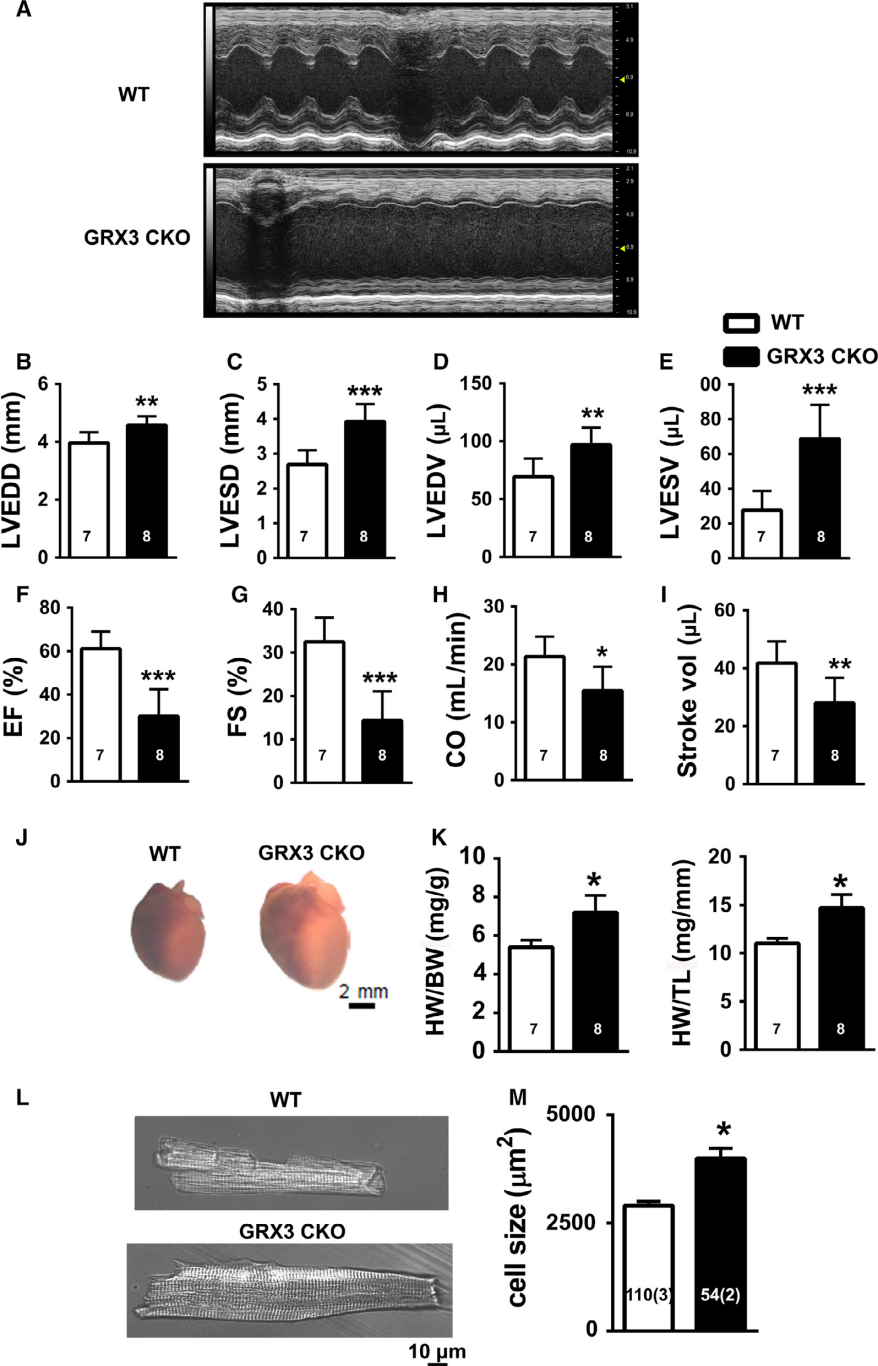
Cardiac hypertrophy and heart failure in conditional knockout (CKO) mice. (A) Representative M‐mode echocardiography tracings of 12‐month‐old control and Grx3 CKO mice showing left ventricular (LV) dilation and impaired contractility. (B–I) Quantification of echo tracings revealed (B–C) increased left ventricular end‐diastolic (LVEDD) and end‐systolic diameters (LVESD), (D–E) increased left ventricular end‐diastolic (LVEDV) and end‐systolic diameters (LVESV, (F–G) reduced ejection fraction (EF) and fraction shortening (FS), (H–I) reduced cardiac output (CO) and stroke volume (SV). (J–K) Postmortem analysis revealed (J) hypertrophied hearts in Grx3 CKO mice, (K) increased heart weight‐to‐body weight (HW/BW) ratios and HW‐to‐tibia length (HW/TL) ratios. Student's *t* test, *n* = 7–8 (7 littermates and 8 CKO mice 0), *P < 0.05, **P < 0.01, and ***P < 0.001 indicate a significance between CKO and littermate controls. (L) Cardiac myocytes isolated from Grx3 CKO mice were hypertrophied, Bars = 10 *μ*m, (M), as evidenced by the cell size analysis. Student's *t* test, *P < 0.05 indicates a significance between CKO and littermate controls. Numbers indicate analyzed cells and mice (in parentheses).

At 12 months of age, there was no difference of body weight between Grx3 CKO mice and littermate controls (38.6 ± 2.2 vs. 41.9 ± 2.1; *P* = 0.304). However, echocardiographic analysis revealed significant LV dysfunction in Grx3 CKO (Fig. 3A). LV end‐systolic diameter (LVESD) and end‐diastolic diameter (LVEDD) were significantly increased in Grx3 CKO hearts (LVESD: 3.93 ± 0.5; LVEDD: 4.58 ± 0.3) compared to littermate controls (LVESD: 2.68 ± 0.4; LVEDD: 3.96 ± 0.4; *P *<* *0.01, *P* < 0.001) (Fig. 3B–C). Accordingly, both LV end‐systolic volume (LVESV) and end‐diastolic volume (LVEDV) were significantly increased in Grx3 CKO hearts (LVEDV: 97.1 ± 14.7; LVESV: 68.75 ± 19.5) compared to littermate controls (LVEDV: 69.3 ± 5.9; LVESV: 27.6 ± 11.0; *P *<* *0.01, *P *<* *0.001) (Fig. 3D–E). Fractional shortening and ejection fraction were significantly decreased in Grx3 CKO mice (EF: 30.1 ± 2.4; FS: 14.4 ± 6.8) versus littermate control mice (EF: 61.1 ± 8.0; FS: 32.5 ± 5.6; *P *<* *0.001) (Fig. 3F–G), along with decreased cardiac output (CO) and stroke volume (Fig. 3H–I).

Furthermore, gross morphological analysis revealed enlargement of Grx3 CKO hearts compared to littermate controls (Fig. 3J). In agreement with this, heart‐to‐body weight and heart weight‐to‐tibial length ratios were significantly increased in Grx3 CKO mice compared to littermate controls (Fig. 3K).

Finally, examinations of isolated cardiomyocytes from Grx3 CKO and littermate control mice revealed an increased size of Grx3 CKO myocytes (3992.3 ± 230.7 *μ*m^2^) compared to littermate controls (2903.9 ± 98.3 *μ*m^2^, *P *<* *0.05) (Fig. 3L–M). Together, these results indicate a significant hypertrophic response and heart failure in 12‐month‐old Grx3 CKO mice.

### Enhanced ROS production in Grx3 CKO hearts

**Figure 4:**
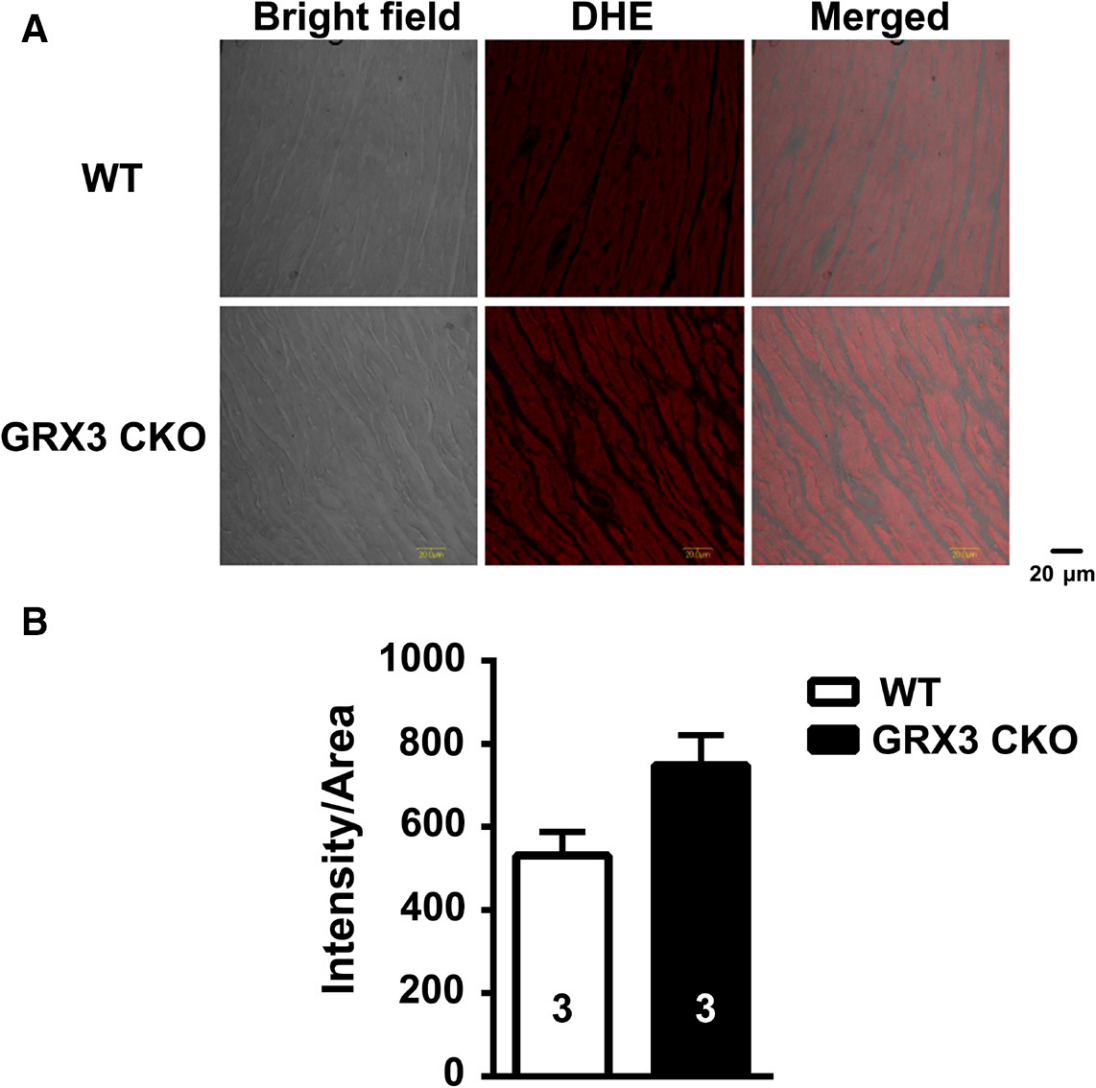
Grx3 deletion enhanced reactive oxygen species (ROS) production and caused cardiac fibrosis in cardiac sections of Grx3 conditional knockout (CKO) mice. (A) Representative images showing DHE fluorescence indicative of elevated ROS levels in cardiac sections of Grx3 CKO mice. Bars = 20 *μ*m. (B) Quantification of signal intensity per area revealing increased ROS levels in cardiac sections from Grx3 CKO mice compared to littermate controls. Student's *t* test, *n* = 3, *P < 0.05 indicates the significance versus controls.

**Figure 5:**
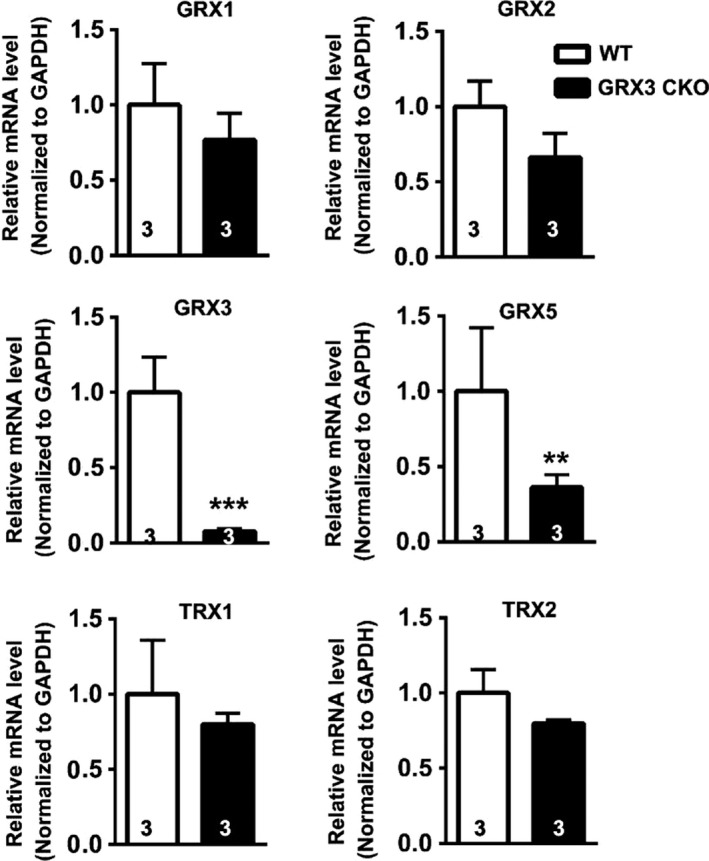
*Grx* and *Trx* gene expression in the Grx3 conditional knockout (CKO) heart. Quantitative polymerase chain reaction (q‐PCR) analysis indicates that *Grx5* mRNA levels were reduced in the Grx3 CKO heart, while other *Grx* and *Trx* gene expression was not altered. Student's *t* test, *n* = 3, **P < 0.01 and ***P < 0.001 indicate the significance versus controls.

To determine whether the loss of Grx3 affects the redox state in the heart, histological analysis of LV sections from Grx3 CKO and littermate control mice was conducted. Sections were stained with dihydroethidium (DHE) to detect ROS levels in the LV cardiomyocytes. Grx3 CKO hearts exhibited a significant increase in DHE‐stained signals (Fig. 4A), indicating increased ROS production in comparison to littermate control hearts (Fig. 4B), suggesting that deletion of Grx3 induces oxidative stress in the heart. To determine if the disruption of Grx3 could alter the expression of other members of *Grx* and *Trx* genes in the CKO hearts, q‐PCR analysis was conducted and showed that only Grx5, a mitochondrial Grx isoform, displayed significantly reduced mRNA levels, while the expression of other major Grx and Trx isoforms was not changed (Fig. 5).

### Altered Ca handling in Grx3 CKO hearts

**Figure 6:**
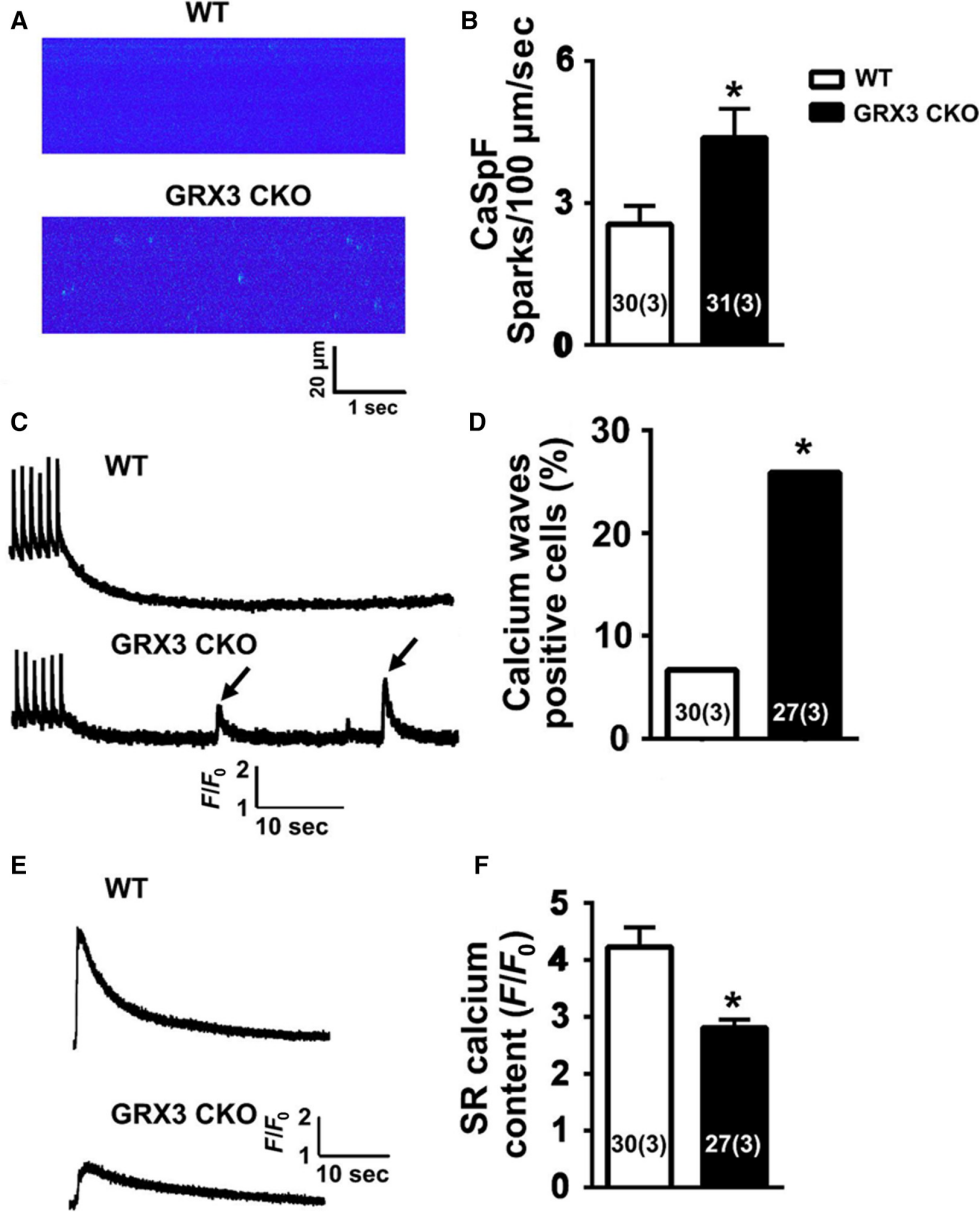
Abnormal intracellular Ca^2+^ handling in myocytes from Grx3 conditional knockout (CKO) mice. (A) Representative line‐scan images of cardiomyocytes from 12‐month‐old mice showing increased Ca^2+^ spark frequency in Grx3 CKO mice. (B) Summary data showing Ca^2+^ spark frequencies following 1‐Hz pacing train. (C) Representative tracings showing spontaneous Ca^2+^ waves in Grx3 CKO mice (arrowheads). (D) Quantification of the percentage of myocytes with spontaneous Ca^2+^ waves. (E) Representative tracings of caffeine‐induced sarcoplasmic reticulum (SR) Ca^2+^ release, which enables the measurement of SR Ca^2+^ content. (F) Quantification of the SR Ca^2+^ content. Numbers in the bar graph indicate the cell number (mouse number). Student's *t* test, *P < 0.05 indicates the significance versus controls. Numbers indicate analyzed cells and mice (in parentheses).

Altered levels of ROS have been shown to affect intracellular Ca^2+^ handling (Wang et al. 2018). To test the effect of Grx3 deficiency on SR Ca^2+^ leak in the heart, Ca^2+^ imaging studies were performed on ventricular myocytes isolated from 12‐month‐old Grx3 CKO and littermate controls. Isolated ventricular myocytes were subjected to a 1‐Hz pacing to obtain steady‐state Ca^2+^ cycling, after which pacing was stopped and the incidence of Ca^2+^ sparks was documented. Representative line‐scan images (Fig. 6A) and bar graphs containing summary data (Fig. 6B) revealed an increased frequency of Ca^2+^ sparks in myocytes from Grx3 CKO mice (4.4 ± 0.6 sparks/100 *μ*m/sec) compared to littermate controls (2.6 ± 0.4 sparks/100 *μ*m/sec; *P *<* *0.05), indicating an enhanced SR Ca^2+^ leak in hearts of Grx3 KO mice. In addition, there was a significant increase of spontaneous Ca^2+^ waves in cardiomyocytes from Grx3 CKO hearts compared to littermate controls (Fig. 6C–D), indicating an increased likelihood of unstimulated contractions and elevated cytosolic Ca^2+^. In contrast, the SR Ca^2+^ content of Grx3 CKO mice showed a significant decrease, probably due to the higher Ca^2+^ leak in Grx3 CKO cardiomyocytes (Fig. 6E–F).

### Loss of Grx3 affects the function of cardiomyocytes from young‐aged mice

**Figure 7:**
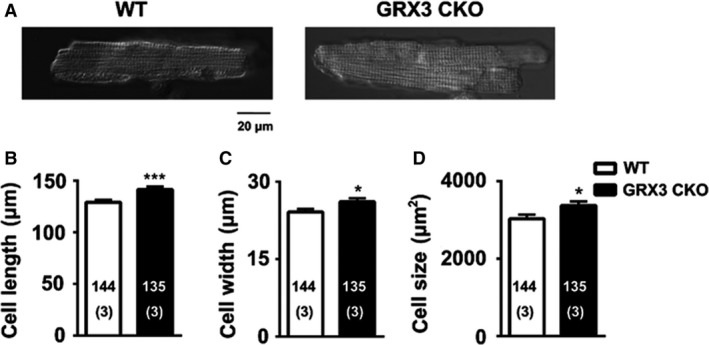
Increased myocyte size in Grx3 conditional knockout (CKO) mice at the age of 3 months. (A) Representative images of ventricular myocytes isolated from Grx3 CKO mice and littermate controls. Bars = 20 *μ*m. Summary data showing increased myocyte length (B), cell width (C), and cell size (D) in Grx3 CKO mice. Student's *t* test, *P < 0.05 indicates the significance versus controls. Numbers indicate analyzed cells and mice (in parentheses).

While there is no difference in gross heart morphology and cardiac function of Grx3 CKO and their littermate mice at 3 months of age (Table 1), it is not clear whether the Grx3 deletion would result in cellular changes at young‐aged mice. To test this, LV myocytes were isolated from 3‐month‐old Grx3 CKO mice and littermate controls (Fig. 7A), which revealed significantly larger myocyte length (Fig. 7B), width (Fig. 7C), and total cell size (Grx3 CKO 3372.6 ± 105.7 vs. littermate controls 3028.5 ± 108.7; *P *<* *0.05) (Fig. 7B–D).

**Figure 8:**
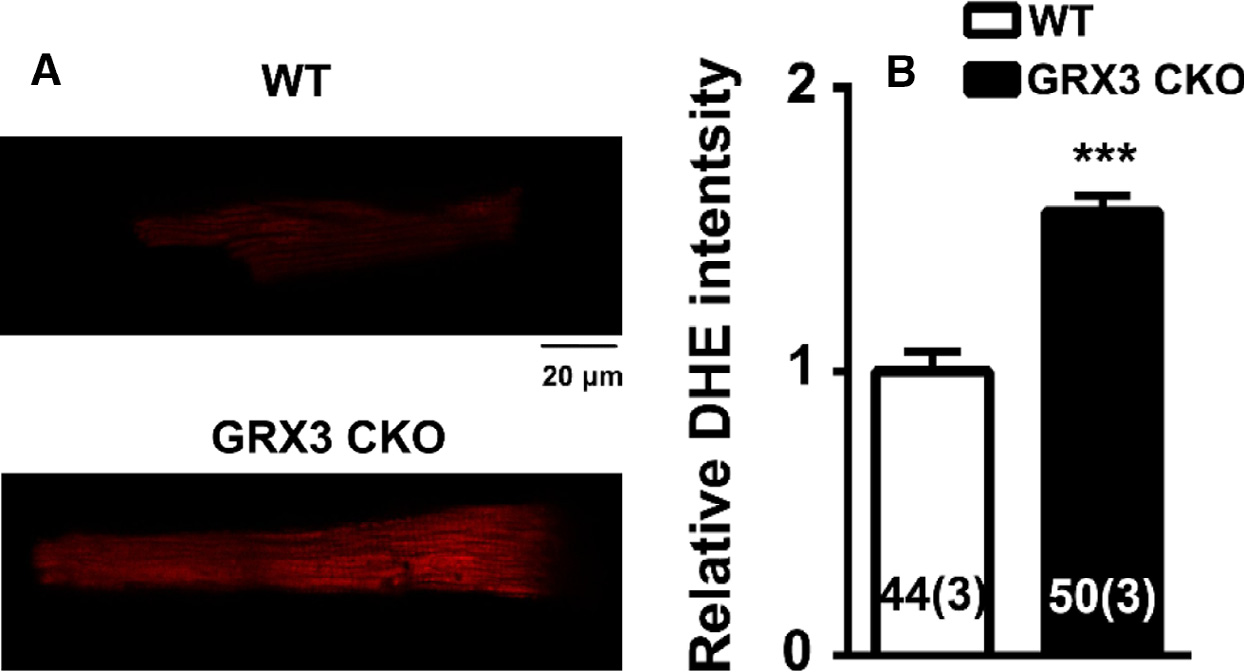
Increased reactive oxygen species (ROS) production in myocytes from Grx3 conditional knockout (CKO) mice. (A) Representative images showing DHE fluorescence revealing ROS levels in ventricular myocytes isolated from Grx3 CKO mice and littermate controls. Bars = 20 *μ*m. (B) Quantification of signal intensity revealing increased ROS levels in Grx3 knockout (KO) myocytes at the age of 3 months compared to littermate controls. Student's *t* test, *P < 0.05 indicates the significance versus controls. Numbers indicate analyzed cells and mice (in parentheses).

The DHE staining of LV myocytes isolated from Grx3 CKO and littermate controls demonstrated a significantly increased ROS production in Grx3 CKO myocytes compared to myocytes from littermate control mice (Fig. 8). These measurements demonstrate that Grx3 deficiency affects redox homeostasis in myocytes of 3‐month‐old mice.

**Figure 9:**
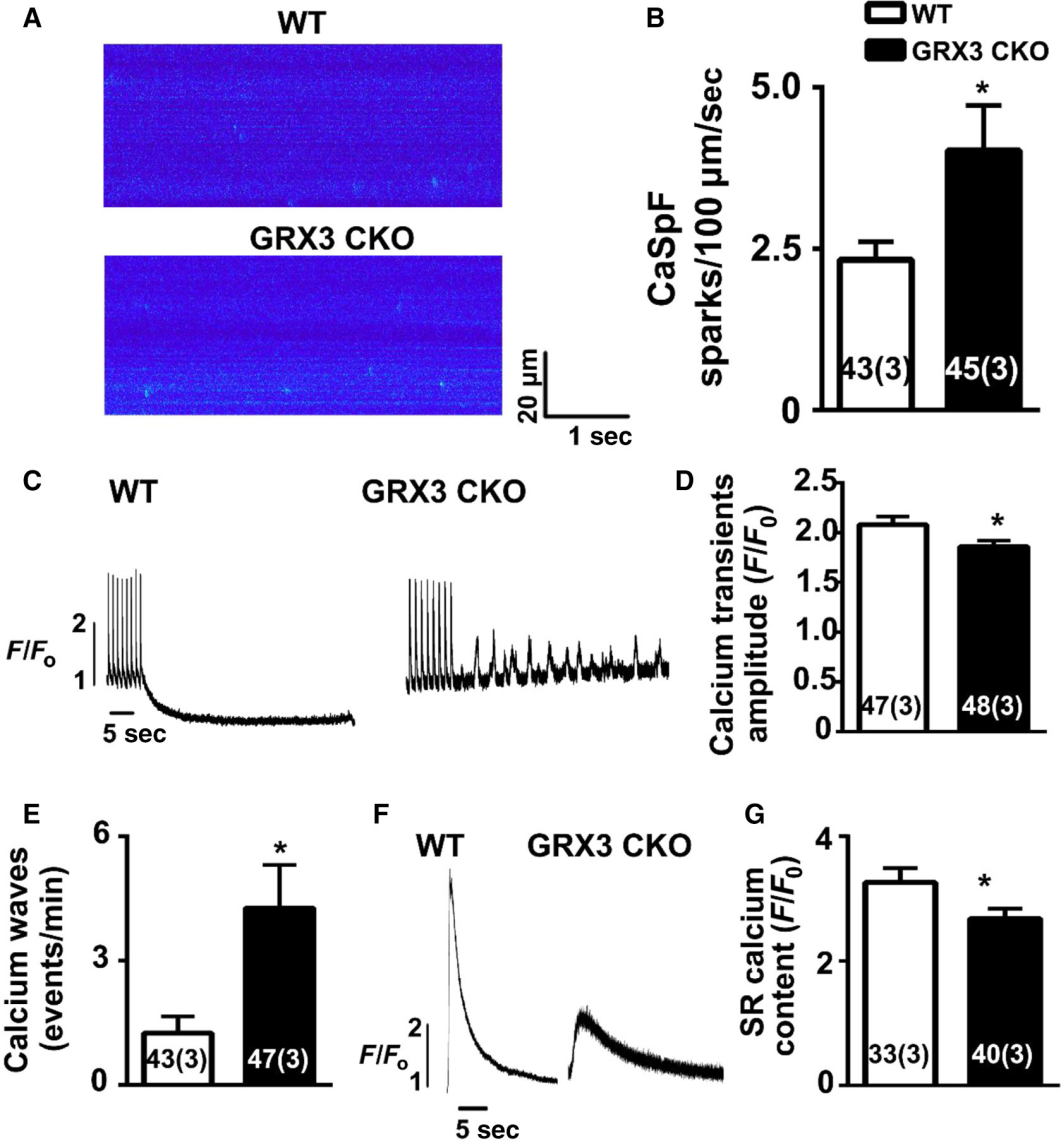
Increased calcium spark frequency, Ca^2+^waves, and decreased sarcoplasmic reticulum (SR) Ca^2+^ load in Grx3 deletion cardiomyocytes at 3 months of age. (A) Representative confocal images and (B) summary data showing increased Ca^2+^ spark frequency in Grx3 conditional knockout (CKO) myocytes compared to control cells (B). (C) Representative Ca^2+^ wave tracings in myocytes from Grx3 CKO mice and littermate controls. (D) Summary data of Ca^2+^ waves in control and Grx3 CKO myocytes. (E) Summary data of Ca^2+^ transient amplitude. (F) Representative tracings of caffeine‐induced SR Ca^2+^ release. (G) Summary data of SR Ca^2+^ content. Numbers in bar graph indicate the cell number (mouse number). Student's *t* test, *P < 0.05 indicates the significance versus controls. Numbers indicate analyzed cells and mice (in parentheses).

Finally, Ca^2+^ imaging studies were performed on ventricular myocytes isolated from 3‐month‐old Grx3 CKO mice. Myocytes from Grx3 CKO mice exhibited significantly increased frequencies of Ca^2+^ sparks compared to littermate control myocytes (Grx3 CKO 4.0 ± 0.7 vs. control 2.3 ± 0.3; *P *<* *0.05) (Fig. 9A and B). The Ca^2+^ transient amplitude was reduced in myocytes from Grx3 CKO mice (1.86 ± 0.06) compared with littermate controls (2.08 ± 0.07; *p *<* *0.05) (Fig. 9C‐D). The frequency of spontaneous SR Ca^2+^ waves following a pacing train was increased in myocytes from Grx3 CKO mice (4.27 ± 1.04) compared with littermate controls (1.25 ± 0.4; *P *<* *0.05) (Fig. 9C and E). Measurement of the SR Ca^2+^ store content using a caffeine dump protocol (Fig. 9F) revealed a reduction in SR Ca^2+^ content in Grx3 CKO mice (2.68 ± 0.16) compared to littermate controls (3.26 ± 0.23; *P *<* *0.05) (Fig. 9G).

These findings suggest that Grx3 deletion in cardiomyocytes alters both ROS levels and intracellular Ca^2+^ handling, which precedes the development of adverse cardiac remodeling associate with heart failure development in these Grx3 CKO mice at a later age.

## Discussion

This study of cardiomyocyte‐specific Grx3 CKO mice revealed that loss of Grx3 causes the development of cardiac hypertrophy and heart failure due to altered both redox homeostasis and SR Ca^2+^ cycling in cardiomyocytes.

Grx3 is ubiquitously expressed in many tissues and organs of mice (Cheng et al. 2011), and its expression is upregulated in muscle cells when exposed to hydrogen peroxide (Cheng et al. 2011). Our previous study revealed that the expression of Grx3 in mouse mammary glands is developmentally regulated (Pham et al. 2017). The current work shows that both Grx3 mRNA expression and protein levels of Grx3 decline in the heart in mice during aging (Fig. 1). The downregulation of Grx3 expression is correlated with an increase in fetal gene expression in the old mouse heart (Fig. 1A). Given that upregulated expression of fetal genes is related to the decline of cardiac function in the aging heart or under cardiac hypertrophy (Molkentin and Dorn 2001; Sun et al. 2015), the levels of Grx3 in the heart may be tightly associated with maintenance of cardiac function during mouse aging or under pathological conditions. In support of this notion, Grx3 has been showed to be induced by hypertrophic agonist endothelin‐1 (ET‐1) and phenylephrine (PE) or following transverse aortic constriction (TAC)‐mediated cardiac hypertrophy (Jeong et al. 2006). Furthermore, a decline of Grx3 expression has been found in human patients with dilated cardiomyopathy and mice with postischemic heart failure (Barth et al. 2006; Lachtermacher et al. 2010). How expression of Grx3 is modulated in the heart during aging and/or under pathophysiological conditions remains to be further investigated.

By crossing Grx3^*flox/flox*^ mice with the *αMhc*‐Cre transgenic line, Grx3 was specifically deleted in the heart, while the expression in other organs/tissues was unaffected (Fig. 2A). Due to the developmentally regulated expression of *αMhc*‐Cre, the deletion of Grx3 expression was incomplete until adulthood (Fig. 2B–E), suggesting a minimal effect on growth and development of neonatal cardiomyocytes in this mouse model. Our results indicate that the absence of Grx3 in the heart does not impair cardiac function in young mice up to 5 months of age. However, in middle‐aged mice (i.e., 12 months), disruption of Grx3 expression led to a hypertrophic response with enlargement of individual myocytes and a steep decline in cardiac function as a result of enhanced ROS production in myocytes. These findings indicate that Grx3 plays a critical role in protecting cardiomyocytes from chronic oxidative damage. Oxidative stress has been shown to be increased in heart failure and contributes to its development and progression (Tsutsui et al. 2011). Our findings suggest that increased ROS production and oxidative stress in Grx3‐deficient cardiomyocytes might be a primary cause to impair cardiac function as the ROS starts accumulating in young mice prior to the decline of cardiac function (Fig. 8). While the mechanisms by which Grx3 regulates ROS and redox homeostasis in cardiomyocytes remain to be fully investigated, we infer that Grx3 could regulate this process with multiple mechanisms. First, Grx3 is an iron–sulfur‐binding protein shown to regulate cellular iron homeostasis (Haunhorst et al. 2010, 2013). Deletion of Grx3 results in iron accumulation in the cell, which could generate cellular ROS through the Fenton chemical reaction. Furthermore, the absence of Grx3 could impair Fe–S cluster assembly that is required for many mitochondrial metabolic enzymes and nuclear proteins (Haunhorst et al. 2013; Stehling et al. 2013) and may increase mitochondrial ROS production. Interestingly, in Grx3‐deficient cardiomyocytes, only expression of Grx5, a mitochondrial Grx involved in iron–sulfur biosynthesis, was significantly decreased (Fig. 5), supporting the notion that mitochondrial function might be impaired in Grx3 CKO heart. In a previous study, the deletion of yeast Grx3 impaired mitochondrial iron homeostasis and Fe–S cluster biogenesis (Muhlenhoff et al. 2010). In mammalian cells, whether reduction of Grx5 in myocytes is part of dysregulation of mitochondrial function (iron regulation and Fe–S cluster biogenesis) caused by Grx3 deletion is still unknown, which warrants future investigation. Second, Grx3 can interact with PKC zeta and inhibit its kinase activity in cardiomyocytes (Oh et al. 2012). Previous studies revealed that PKC zeta can regulate NOX2 activity and control NOX‐mediated ROS production through phosphorylation of p47^*phox*^ (Fontayne et al. 2002). Therefore, deletion of Grx3 could activate PKC zeta activity and NOX‐mediated ROS production in hearts of Grx3 CKO mice. Furthermore, our previous investigation demonstrates that Grx3/Txnl2 can control glutathione metabolism in cancer cells by regulating the NF‐*κ*B signaling pathway (Qu et al. 2011). It is possible that Grx3 could control redox homeostasis in the cardiomyocytes through a transcriptional mechanism. Nevertheless, the precise mechanism underlying Grx3‐mediated ROS production in the cardiomyocytes remains to be investigated in future studies.

Loss of Grx3 in cardiomyocytes significantly affects SR Ca^2+^ release, resulting in decreased SR Ca^2+^ uptake, which in turn contributes to the decline in cardiac function in older mice. This accelerated SR Ca^2+^ leak and decreased SR Ca^2+^ storage occur in cardiomyocytes of young mice prior to the development of cardiac hypertrophy and heart failure (Fig. 9), suggesting that alteration of Ca^2+^ handling could also be the contributing factor to development of cardiac hypertrophy and failure in Grx3 CKO mice. Reduction of SR Ca^2+^ storage in Grx3 CKO cardiomyocytes is likely caused by a decreased SERCA activity. Previous studies indicate that Grx3/PICOT increases SERCA activity by modulating phospholamban (PLB) activity, because phosphorylation of PLB at threonine 17 is impaired in Grx3 haploinsufficient myocytes (Cha et al. 2008). However, whether this regulatory process is ROS dependent remains to be determined.

In our current study, we demonstrated that deletion of Grx3 caused ROS accumulation and dysregulation of Ca^2+^ handling in the heart. Both processes could independently account for cardiac dysfunction (hypertrophy and heart failure) in Grx3 CKO mice. Excess ROS can modify and/or even damage macromolecules in the cell (Droge 2002). It has been shown that oxidation of ion channels and Ca^2+^ transporters alters Ca^2+^ handing, which causes dysregulation of excitation–contraction coupling in cardiomyocytes (Köhler et al. 2014) and oxidized cardiac ryanodine receptor (RyR2) contributes to sarcoplasmic reticulum Ca^2+^ leak in chronic heart failure (Terentyev et al. 2008). Therefore, there is possibility that Ca^2+^ handling problem could be caused by ROS‐mediated oxidative stress in Grx3 CKO mice, which warrants future investigation.

Previous studies indicate that Grx3, especially the C‐terminal region of Grx3, can physically interact with a muscle LIM protein (MLP) to prevent the formation of the MLP–calcineurin complex, and thus blocking calcineurin–NFAT signaling associated with the development of cardiac hypertrophy under pressure overload (Jeong et al. 2008). Therefore, the absence of Grx3 in cardiomyocytes might activate the MLP–calcineurin–NFAT pathway that leads to cardiac hypertrophy and heart failure as seen in our Grx3 CKO mouse model. Whether this regulation is redox‐dependent remains to be determined.

In conclusion, our findings support the hypothesis that Grx3 is an important factor in regulating cardiac hypertrophy and heart failure by controlling both cellular redox homeostasis and Ca^2+^ handling in the heart.

## Conflict of Interest

The authors have nothing to disclose.
